# Redesign of a rapid, low‐cost HPV typing assay to support risk‐based cervical screening and management

**DOI:** 10.1002/ijc.34151

**Published:** 2022-06-24

**Authors:** Kanan T. Desai, Clement A. Adepiti, Mark Schiffman, Didem Egemen, Julia C. Gage, Nicolas Wentzensen, Silvia de Sanjose, Robert D. Burk, Kayode O. Ajenifuja

**Affiliations:** ^1^ Clinical Epidemiology Unit, Clinical Genetic Branch, Division of Cancer Epidemiology and Genetics National Cancer Institute Rockville Maryland USA; ^2^ Department of Obstetrics, Gynecology, and Perinatology The Obafemi Awolowo University Ile‐Ife Osun State Nigeria; ^3^ ISGlobal Barcelona Spain; ^4^ Albert Einstein Cancer Center Albert Einstein College of Medicine New York New York USA

**Keywords:** cervical screening, HPV genotyping, isothermal amplification, risk‐based management

## Abstract

Accelerated cervical cancer control will require widespread human papillomavirus (HPV) vaccination and screening. For screening, sensitive HPV testing with an option of self‐collection is increasingly desirable. HPV typing predicts risk of precancer/cancer, which could be useful in management, but most current typing assays are expensive and/or complicated. An existing 15‐type isothermal amplification assay (AmpFire, Atila Biosystems, USA) was redesigned as a 13‐type assay (ScreenFire) for public health use. The redesigned assay groups HPV types into four channels with differential cervical cancer risk: (a) HPV16, (b) HPV18/45, (c) HPV31/33/35/52/58 and (d) HPV39/51/56/59/68. Since the assay will be most useful in resource‐limited settings, we chose a stratified random sample of 453 provider‐collected samples from a population‐based screening study in rural Nigeria that had been initially tested with MY09‐MY11‐based PCR with oligonucleotide hybridization genotyping. Frozen residual specimens were masked and retested at Atila Biosystems. Agreement on positivity between ScreenFire and prior PCR testing was very high for each of the channels. When we simulated intended use, that is, a hierarchical result in order of clinical importance of the type groups (HPV16 > 18/45 > 31/33/35/52/58 > 39/51/56/59/68), the weighted kappa for ScreenFire vs PCR was 0.90 (95% CI: 0.86‐0.93). The ScreenFire assay is mobile, relatively simple, rapid (results within 20‐60 minutes) and agrees well with reference testing particularly for the HPV types of greatest carcinogenic risk. If confirmed, ScreenFire or similar isothermal amplification assays could be useful as part of risk‐based screening and management.

## Graphical abstract

What's new?

Due to cost and perceived complexity, most existing human papillomavirus (HPV) assays for cervical cancer screening are designed to yield a pooled result for the carcinogenic HPV types. Here, to promote rapid, affordable and risk‐based cervical screening, an existing isothermal DNA amplification test was redesigned to group the carcinogenic HPV types into four channels based on clinical importance (HPV16; HPV18/45; HPV 31/33/35/52/58 and HPV 39/51/56/59/68). In masked retesting of 453 Nigerian specimens, the new ScreenFire assay showed good‐to‐excellent type group agreement with prior PCR testing. When validated, the redesigned test could support risk‐based screening in resource‐limited settings.

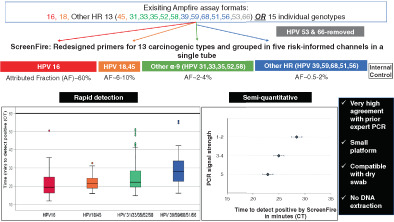

**Abbreviations**
ANOVA: analysis of variance
CE: Conformité Européenne
CI: confidence interval
CIN2+: cervical intraepithelial neoplasia grade 2+
COVID‐19: Coronavirus Disease of 2019
ct: cycle threshold
HPV: human papillomavirus
HR: High‐risk
IARC: International Agency for Research on Cancer
IQR: interquartile range
IRB: Institutional Review Boards
LBC: liquid‐based cytology
NCI: National Cancer Institute
PCR: polymerase chain reaction
SPSS: Statistical Package for Social Sciences
WHO: World Health Organization

## 1 INTRODUCTION

Cervical cancer incidence and mortality are highest in lower‐resource regions of the world.^1^ The World Health Organization (WHO)'s global call for eliminating cervical cancer relies on human papillomavirus (HPV) vaccination, two rounds of HPV based screening in mid‐adulthood and treatment of cervical precancers and cancers.^2^ Given the necessary and causal role of HPV in cervical carcinogenesis,^3^ sensitive HPV‐test based screening is now recognized as the preferred screening test for cervical cancer prevention worldwide.^4^, ^5^, ^6^

Nevertheless, HPV infections are too common and benign in many high‐risk regions to treat all infected women.^7^, ^8^ Most HPV infections clear rapidly within 1 to 2 years of initial detection.^9^ ^,^ ^10^ Only persistent HPV infections, in the absence of clearance, progress to precancer and cancer.^9^ The risk of progression, given persistence, depends on HPV type and varies substantially even among the 13 high‐risk (HR) HPV types defined as human carcinogens (ie, Group 1) or probable carcinogens (ie, Group 2A) by the International Agency on Research for Cancer (IARC).^11^ Evidence to date suggests that at least four distinct risk categories exist, including: (a) HPV16 (species alpha‐9) with the highest risk of cervical cancer, causes ~60% of squamous cancers, (b) HPV18 and HPV45 (species alpha‐7) with an intermediate risk of cervical cancer, cause ~15% of squamous cancers but together with HPV16 account for >90% of adenocarcinomas,^12^ (c) other HPV16‐related alpha‐9 types, namely, HPV31, HPV33, HPV35, HPV52 and HPV58, with intermediate risk of cancer, cause another 15% of squamous cancers and (d) other HR types, namely, HPV39, HPV59, HPV68 (species alpha‐7), HPV56 (species alpha‐6) and HPV51 (species alpha‐5), with low risk of cervical cancer, account for only ~5% of squamous cancers but represent a quarter of HR13 HPV infections.^9^, ^13^, ^14^, ^15^, ^16^, ^17^, ^18^ Risk‐based extended HPV typing, if incorporated with minimal additional cost into primary HPV screening, could provide risk stratification for triage of HPV‐positive women in a single step.^19^

Due to cost and perceived complexity, most existing HPV assays are designed to yield a pooled result for the carcinogenic HPV types with no or only limited genotyping (mainly of HPV16 and HPV18), obscuring the finer risk‐stratifications within the other HR HPV types (eg, HPV16‐related alpha‐9 types pose a substantially greater risk than the “other HR” types). Moreover, many existing assays also include HPV66 and a few include HPV53 (species alpha‐6). The latest version of the IARC monograph, rectifying the mistake of the previous versions, reclassifies HPV66 along with HPV53 as only possibly and rarely carcinogenic to humans (ie, Group 2B).^11^ HPV66 and HPV53 are relatively common in the general population and frequently cause high‐grade appearing lesions but rarely cause cancer.^20^ Thus, including these marginally carcinogenic HPV types in a screening assay is likely to harm public health due to a decreased specificity and positive predictive value without appreciable gain in sensitivity and negative predictive value for screening to prevent cervical cancer.^20^, ^21^

Additionally, HPV assays need to be rapid, low‐cost and practical for scale‐up in cervical cancer screening programs in resource‐limited settings. An isothermal amplification technology‐based AmpFire HPV assay (Atila Biosystems, Inc., Mountain View, California) shows promise in this regard.^22^ The assay in its two current formats offers (a) multiplex detection in a single tube of 15 HPV types (ie, including HPV53 and HPV66) with separate detection of types 16/18 or (b) full genotyping of 15 individual types in four tubes. The assays are Conformité Européenne (CE) marked^23^ and previously demonstrated to have satisfactory performance in analytical^24^ and clinical validation.^23^

We collaborated with the scientists at Atila Biosystems to redesign the AmpFire HPV assay into a single‐step 13‐type assay providing risk‐based, extended HPV genotyping in four channels in a single tube as follows: (a) HPV16, (b) HPV18/45, (c) HPV31/33/35/52/58 and (d) HPV39/51/56/59/68. These efforts were undertaken for public health benefit. The current article aims to independently validate the redesigned AmpFire HPV assay (ScreenFire HPV RS hereafter referred to as ScreenFire) compared against a well‐validated MY09‐MY11‐based polymerase chain reaction (PCR) assay with genotyping.

## 2 MATERIALS AND METHODS

### 2.1 Study design and population

Our study utilized 1339 residual provider‐collected cervical samples from a population‐based screening study of 16 to 88 year‐old (mean [SD] = 44.0 [15.8]) women in rural Nigeria. Project Itoju was conducted during 2009 to 2010; residual specimens were stored frozen in Preservcyt solution (Hologic, Marlborough, Massachusetts). The study methodology has been described in detail previously.^25^ Briefly, ~1420 eligible women (not pregnant, sexually experienced and without hysterectomy, 15+ years old and living in the house for more than 3 months) from randomly selected houses in the village of Irun in Nigeria were invited and attended the screening clinic and provided informed consent to be enrolled in the study. At the screening visit, locally trained nurses performed cervical exams and collected cervical cell sample in Preservcyt (Hologic, Marlborough, Massachusetts) using a broom‐type device and endocervical brush for liquid‐based cytology (LBC) and HPV testing.

One milliliter aliquots of the frozen residual cytology samples were tested in the United States (Albert Einstein Cancer Center) for HPV using a AmpliTaq Gold MY09‐MY11 PCR‐based test that included additional type‐specific primers, and primers to amplify a cellular beta‐globin fragment as an internal control (IC) for amplification as previously described.^26^ PCR products were genotyped with dot‐blot hybridization using type‐specific probes for 13 HR and >20 other HPV types, using a numeric measure of signal strength (1‐5 from lowest to highest) as a validated semiqualitative measure of viral load.^27^ Additionally, samples were also tested with an investigational Luminex assay for HPV16 and HPV18 DNA, which we considered only in supplemental analyses.

Of the 1339 such residual samples, valid PCR results were available for 1305 samples. A small set (n = 8) of typed samples positive for only low‐risk HPV types was unblinded and provided to the Atila Biosystems laboratory for the modification of the AmpFire assay chemistry. Of the remaining 1297 masked samples with valid results, we chose all 356 samples that had tested positive for any HPV types, including low‐risk types, by either PCR or HPV16/18 Luminex, and 100 randomly selected specimens testing HPV negative for all HPV types including low‐risk, by both PCR and Luminex. These specimens were provided to Atila BioSystems with masked labeling and retested there with the ScreenFire HPV assay.

The first masked comparison generated fair to good agreement (data not shown). There was evidence of competition for reagents in the presence of multiple HPV infections, and slightly diminished sensitivity for some types. We provided the summary results to the Atila Biosystems team and re‐randomized the specimens under NCI direct supervision to permit another valid masked testing round. After primer redesign and reagent optimization were completed, the Atila Biosystems team “locked” their new format, which emphasized more sensitive detection particularly of HPV16 and HPV18/45. The masked testing of the specimen set was then completely repeated. This article reports the independent analysis of results from the second round of testing.

### 2.2 ScreenFire HPV Test

The assay is intended to be performed on “dry swabs”; thus, centrifugation was needed to process the 0.5 mL of cervical cell suspension in Preservcyt. Specifically, the suspension was transferred to a 1.5 mL tube for centrifugation at maximum speed for 20 minutes and the supernatant was discarded, followed by the addition of 50 μL of ×1 lysis buffer and vortexing thoroughly to resuspend the cell pellet. The resuspended cell pellets were then transferred to 96‐well PCR plates for incubation at 95°C for 15 minutes, cooled at room temperature and briefly spun. After this initial sample preparation, 5 μL of this prepared specimen was mixed with 20 μL of freshly prepared master mix (including reaction mix and primer mix) into a 96‐well PCR plate using hand pipetting. Additionally, positive and negative template controls were included in each 96‐well plate as well. Next, the plates were sealed with an optical compatible film, vortexed gently for mixing the reagents and centrifuged to bring down all liquid to the bottom of the wells. The plates were then loaded into the Bio‐Rad CFX96 real‐time PCR machine on the isothermal program mode run at 1 minute per cycle at 60°C for 60 cycles with fluorescence obtained from CY5 (for HPV16), ROX (for HPV18/45), CY5.5 (for HPV31/33/35/52/58), FAM (for HPV39/51/56/59/68) and HEX (for Human Beta Globin Gene as Internal Control [IC]). A sample was considered positive for an HPV channel if the signal was detected within 60 minutes in the channel, regardless of the signal in the HEX channel. If no signal was detected for any of the four HPV channels within 60 minutes, then a signal was required in the HEX channel for the batch run to be called a valid negative.

### 2.3 Statistical analysis

Valid ScreenFire HPV results were available for 453 of the 456 samples and were included in the analysis. HPV results from PCR were categorized a posteriori into the same four risk groups (ie, HPV16; HPV18/45, HPV31/33/35/52/58 and HPV39/51/56/59/68).

First, HPV group results were considered nonhierarchically, recognizing that a given specimen could test positive for more than one channel. Agreement statistics and McNemar's test for asymmetry were calculated for each of the channels. The analysis accounted for sampling of HPV‐negatives by reweighting to the original sample size for point estimates, but maintained standard errors derived from the subset tested. Box and whisker plots were examined for time to detection (the equivalent of cycle threshold [ct]‐values in PCR assays) of the positive samples for each channel by the ScreenFire HPV assay. Outlier positive samples were identified as the ones with “time to detect positive” of greater than Q3 (third quartile of detection times) + 1.5 times interquartile range (IQR) in minutes. For the samples that tested positive by both the ScreenFire and PCR assay, an association between the time to detect positive in minutes by the ScreenFire assay and the signal strength by PCR assay was examined by Analysis of Variance (ANOVA) and nonparametric ranked test for trend in a pooled analysis combining the channels, including only samples positive by PCR for a single HR13 HPV type and positive by ScreenFire for a single HPV channel (n = 127). The PCR signal strength variable^1^, ^2^, ^3^, ^4^, ^5^ is not strictly linear, in that a signal strength of 5 has no upper bound and on average is particularly strong. For the samples that tested positive by the ScreenFire assay, an association between the time to detect positive HPV and internal control (HEX signal) in minutes by the ScreenFire assay was examined by correlation coefficients in a pooled analysis combining the channels, including only samples positive by ScreenFire for a single HPV channel (n = 165).

Second, the two tests were also compared using risk‐based hierarchical HPV group types, considering that varying risks of cervical cancer associated with different risk groups are usefully interpreted according to the highest‐risk result obtained to permit risk‐based management. Thus, the hierarchical risk‐groups were considered as HPV16 positive, else positive for HPV18 or HPV45 (if HPV16 was not present), else positive for HPV31, HPV33, HPV35, HPV52 or HPV58 (if HPV16, 18 and 45 were not present, etc), else positive for HPV39, HPV51, HPV56, HPV59 or HPV68, else negative. Agreement on positive results and kappa values were calculated for the hierarchical analysis.

As a supplementary analysis, the investigational Luminex testing for HPV16 and HPV18 was examined to look for clues as to the meaning of interassay disagreement. Results from this assay are presented because they revealed interesting patterns but are outside the a priori comparison.

All analyses were conducted using Microsoft Excel, SPSS (statistical package for social sciences) (Build 1.0.0.1089) and R (version 3.6.2).

## 3 RESULTS

### 3.1 Channel by channel analysis

There was good to excellent agreement between the ScreenFire assay and the PCR assay for all the channels on nonhierarchical analyses (Table 1). No major differences in the agreement were observed when the age range was restricted to 25 to 49 years (data not shown). ScreenFire tended to have slightly, nonsignificantly higher positivity than PCR, for all four type group channels. The additional positives by the ScreenFire HPV assay did not show any specific cross‐reactivity patterns with other high‐risk or low‐risk HPV types detected by PCR (data not shown). For the additional positives by the PCR assay, results were limited by small numbers, but four of the five such positives on the HPV39/51/56/59/68 channel were HPV51 positive by PCR.

**TABLE 1:** Nonhierarchical agreement between ScreenFire genotyping channels and type‐specific PCR‐based results (n = 453)

HPV results (ScreenFire/PCR)	+/+ (N)	−/+ (N)^a^	+/− (N)^b^	−/− (N)	Percent agreement on positives (95% confidence interval [CI])	McNemar test (*P*‐value)
HPV16	24	3	8	418	68.6% (53.2%‐84.0%)	.23
HPV18/45	24	1	4	424	82.8% (69.0%‐96.5%)	.37
HPV31/33/35/52/58	111	5	12^c^	325	86.7% (80.8%‐92.6%)	.15
HPV39/51/56/59/68	44	5	13^c^	391	71.0% (59.7%‐82.3%)	.10

^a^ For the ScreenFire negative, PCR positive samples, for the HPV16 channel, the PCR signal strength was 1, 2, 3 for each of three such samples. For the HPV18/45 channel, the PCR type was HPV18 (signal strength 2) for one such sample. For the HPV31/33/35/52/58 channel, for five such samples, the PCR types were HPV31 for two (signal strength 1, 2), HPV52 for one (signal strength 2), HPV58 for two (signal strength 2, 5). For the HPV39/59/68/51/56 channel, for five such samples, the PCR types were HPV51 for three (signal strength 2, 3, 5), HPV51 and 68 for one (signal strength 1 and 2, respectively), HPV56 for one (signal strength 1).

^b^ For the ScreenFire positive, PCR negative samples, for the HPV16, HPV18/45, HPV31/33/35/52/58 and HPV39/51/56/59/69 channels the median time to positive in minutes on AmpFire were 20.8 (IQR: 8.1), 27.7 (IQR: 6.8), 25.1 (IQR: 7.4) and 29.5 (IQR: 9.3), respectively.

^c^ No type‐specific cross‐reactivity pattern detected for more than 20 other low‐risk HPV types detected by PCR.

**FIGURE 1:**
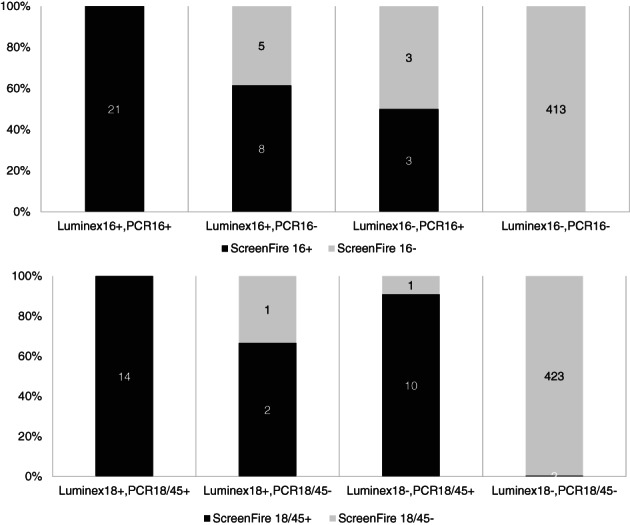
Nonhierarchical agreement between ScreenFire channels for HPV16 and HPV18/45 with type‐specific PCR and investigational Luminex‐based results

Whenever there was an agreement between PCR and the investigational Luminex assay for HPV16, ScreenFire always agreed with them. When there was discrepancy between PCR and Luminex assay (n = 19), ScreenFire agreed with Luminex for 57.9% (11 of 19) times and with PCR for 42.1% (8 of 19) times. Whenever there was an agreement between PCR for HPV18/45 and investigational Luminex assay for HPV18, ScreenFire for HPV 18/45 agreed with them for all but one time. When there was discrepancy between PCR and Luminex assay (n = 14), ScreenFire agreed with Luminex for 21.4% (3 of 14) times and with PCR for 78.6% (11 of 14) times. This is likely due to lack of HPV45 in Luminex assay (Figure 1).

**FIGURE 2:**
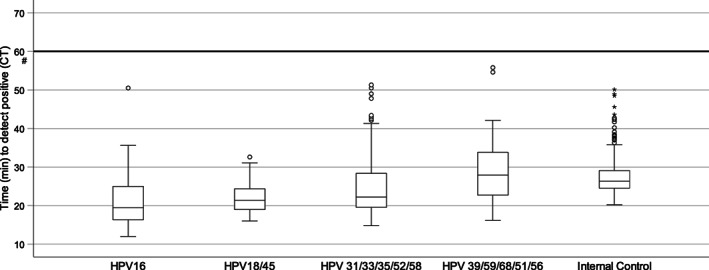
Box‐whisker plot for run time to detection of positive samples for each HPV channel by the ScreenFire HPV test. #Suggested maximum allowed time to detect positive is 60 minutes. ^o^One outlier sample for HPV16 channel was PCR positive for HPV 16 (signal strength of 4, histopathologic CIN3). One outlier sample for HPV18/45 channel was PCR negative (Luminex positive for HPV18). Five of the eight outlier samples for HPV31/33/35/52/58 channel were PCR positive for HPV58 (signal strength 2, 3, 4, 4, 5), one was PCR positive for HPV31 and 58 (signal strength 2 and 3, respectively), one was PCR positive for HPV35 and 52 (signal strength 2 for each, histopathologic CIN3) and one was PCR negative. Two outlier samples for HPV39/59/68/51/56 were PCR positive for HPV56 (signal strength 3, 5)

Despite the maximum allowance of 60 minutes to detect positive results by the ScreenFire assay, for HPV16, HPV18/45 and HPV31/33/35/52/58 channels 50% of positive samples were evident within 25 minutes, and 75% of positive samples were noticeable within 30 minutes (Figure 2). For the HPV39/51/56/59/68 channel, time to positive was slightly longer: 50% of positive samples were evident within 35 minutes, and 75% of positive samples were noticeable within 40 minutes. For the HPV16, HPV18/45 and HPV39/51/56/59/68 channels, only one of 32 (3.1%), one of 28 (3.6%) and two of 57 (3.5%) ScreenFire positive samples, respectively, were outliers (ie, had positive results after 38, 32, 51 minutes respectively for each channel). However, for the HPV31/33/35/52/58 channel, eight of 123 (6.5%) ScreenFire positive samples were outliers (ie, had positive results after 42 minutes) and six of the eight were PCR HPV58 positive (signal strength from PCR ranged from 2 to 5). Overall, out of a total of 32 PCR HPV58 positive samples, two tested negative by the ScreenFire assay in addition to the six relatively delayed positive results (total 8 of 32 or 25%).

**FIGURE 3:**
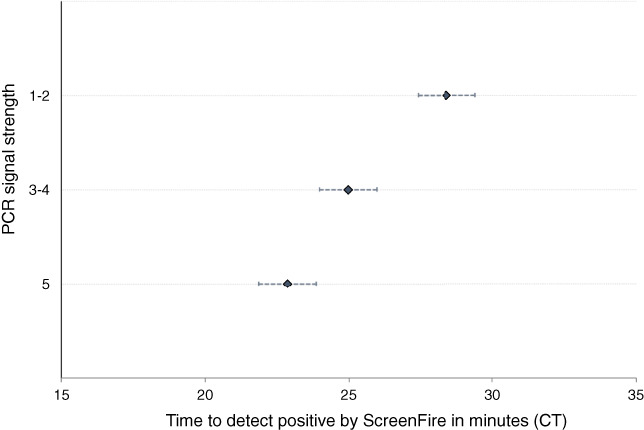
Association between time to detect positive by the ScreenFire HPV test and the signal strength by the PCR test (n = 127). *P* = .023 on ANOVA, *P* < .001 on ranked trend test. [Color figure can be viewed at wileyonlinelibrary.com]

A significant association was found between the time to detect positive samples on the ScreenFire HPV assay and the signal strength by PCR (*P*‐value =.02 on ANOVA, *P*‐value <.001 on ranked trend test) (Figure 3). No significant association was found between the time to detect positive samples on the ScreenFire HPV assay and the presence of single vs multiple channel positive results (*P*‐value >.05 on independent sample *t*‐tests) (data not shown). However, a significant negative correlation was found between the time to detect positive HPV signals and the time to detect positive internal control (HEX) signal on the ScreenFire HPV assay (Pearson's correlation coefficient = (−0.25), *P*‐value =.001; Spearman's correlation coefficient = (−0.38), *P*‐value <.001), suggesting some reagent competition.

### 3.2 Hierarchical analysis

On the hierarchical analysis, which would be useful for risk‐based clinical management of the women, there was an overall 96.8% (95% confidence interval [CI]: 95.9%‐97.8%) agreement between the ScreenFire and PCR assay (unweighted kappa = 0.89, weighted kappa = 0.90, these statistics are obtained by using sampling weights) (Table 2). Overall type‐group agreement restricted to positives was 80.9% (95% CI: 75.7%‐86.2%).

**TABLE 2:** Hierarchical agreement between ScreenFire‐based HPV genotyping channels and type‐specific PCR‐based results, according to HPV risk groups

ScreenFire	PCR						
	HPV16	Else HPV18/45	Else HPV31/33/35/52/58	Else HPV39/51/56/59/68	Else positive for low‐risk HPV types^a^	Else HPV negative for all types	Total for ScreenFire
**HPV16**	**24**	0	2	0	2	4	**32**
**Column %**	**88.9%**	0.0%	2.0%	0.0%	1.3%	3.8%	**7.1%**
**Else HPV18/45**	0	**24**	2	0	1	1	**28**
**Column %**	0.0%	**96.0%**	2.0%	0.0%	0.6%	1.0%	**6.2%**
**Else HPV31/33/35/52/58**	3	1	**90**	2	5	1	**102**
**Column %**	11.1%	4.0%	**90.9%**	4.8%	3.2%	1.0%	**22.5%**
**Else HPV39/51/56/59/68**	0	0	0	**36**	6	2	**44**
**Column %**	0.0%	0.0%	0.0%	**85.7%**	3.8%	1.9%	**9.7%**
**Else HPV negative for high‐risk types**	0	0	5	4	**140**	**98**	**247**
**Column %**	0.0%	0.0%	5.1%	9.5%	**89.7%**	**94.2%**	**54.5%**
**Total for PCR**	**27**	**25**	**99**	**42**	**156**	**104**	**453**
**Column %**	**100.0%**	**100.0%**	**100.0%**	**100.0%**	**100.0%**	**100.0%**	**100.0%**

*Note*: Highlighted in gray are concordant HPV risk group results (n = 453). Unweighted kappa = 0.89 (95% CI: 0.85‐0.92); Weighted kappa = 0.90 (95% CI: 0.86‐0.93); Overall percent agreement = 96.8% (95% CI: 95.9%‐97.8%); Overall agreement on positives = 80.9% (95% CI: 75.7%‐86.2%). These statistics are obtained by using sampling weights. In the study, only 100 of the 941 HPV negatives for all HPV types on both PCR and Luminex are used, therefore in the statistical analyses we are using sampling weights to weight the sample back to the original population.

a Low HPV types detected by PCR are: HPV6, 26, 30, 34, 53, 61, 62, 66, 69, 70, 71, 72, 73, 81, 82, 83, 86, 87, 90 and 106.

Data on valid liquid‐based cytology and histopathology were available for only 420 and 172 samples, respectively. Of these, for the 12 histopathologic high‐grade Cervical intraepithelial neoplasia grade 2+ (CIN2+) cases, 10 were detected by both the ScreenFire and PCR assay, both assays missed one case and the ScreenFire assay picked up one additional CIN2 on the HPV39/51/56/59/68 channel. Nevertheless, both assays were positive for all five CIN3 cases. In fact, out of 36 cytologic or histopathologic high‐grade samples, there was only one discordant result (Table 3).

**TABLE 3:** Agreement between ScreenFire and PCR‐based results for any HR13 types, for most serious histopathologic and/or cytologic results

		HPV results (ScreenFire/PCR)			
		+/+ (N)	−/+ (N)	+/− (N)	−/− (N)
Histopathology (n = 172)	Less than cervical intraepithelial neoplasia (CIN) Grade 2 (<CIN2)	73	2	8	77
	CIN2	5^a^	0	1^b^	1
	CIN3	5	0	0	0
Cytology (n = 420)	Negative (includes within normal limit, atypical squamous cell [ASC], low‐grade squamous intraepithelial lesion [LSIL] including cellular changes)	149	7	20	216
	High‐grade squamous intraepithelial lesion or higher (HSIL+) (includes ASC rule out HSIL, HSIL‐CIN2, HSIL‐CIN3, atypical glandular cell [AGC] favor neoplasia, AGC not otherwise specified [AGC NOS], invasive cancer)	24			4^c^

^a^ One sample positive for HPV16, 58 by PCR was only HPV31/33/35/52/58 channel positive by ScreenFire and had invasive squamous cancer on cytology.

^b^ Positive for HPV39/59/68/51/56 channel.

^c^ Two samples were HSIL CIN3, one sample was ASC rule out HSIL, one sample was AGC NOS.

## 4 DISCUSSION

The ScreenFire HPV assay accurately measured HPV DNA positivity compared to PCR, yielding risk‐based HPV genotype groupings of the 13 carcinogenic types, within 1 hour and usually within a half hour. The validation compared to prior reference PCR testing showed good to excellent agreement for each of the four risk‐based HPV genotyping groups defined by the ScreenFire HPV assay. ScreenFire tended to call slightly more positives than the MY09‐MY11 PCR although they were not statistically significantly different. For HPV16 and HPV18, there was some indication that the extra positives might be true positives if the results of the research Luminex assay are considered to be useful a posteriori for adjudication. There was also some indication of the time to detect positive HPV as an indicator of viral load.

The ScreenFire assay is relatively rapid (combining the run time and ~30 minutes of hands‐on preparation time to run 94 specimens), simple (no batch testing required, can run 1 to 94 samples without product wastage) and less expensive than most other HPV typing tests (~5 USD per sample with scale‐up).^22^, ^23^ Based on evidence from earlier AmpFire versions, it detects HPV (and a human DNA control) from the clinical specimen without a need for DNA extraction. So, it is compatible by design and by apparent analytic sensitivity with dry self‐collected specimens without a need for collection media^28^ or special laboratories.^22^, ^23^ The dry swab can be stored at room temperature for up to 2 weeks and at −20°C for up to a month. The test does require basic pipetting skills.^22^, ^23^ The test reagents are stable at room temperature for 2 weeks, at 4°C for up to a month and at −20°C for a year. In addition, the test platform is portable and the same platform can test for other sexually transmitted infections and Coronavirus Disease of 2019 (COVID‐19).^22^, ^23^

The assay could help to address an identified public health need. In resource‐limited settings, which account for 90% of cervical cancer deaths, vaccine coverage and particularly organized screening are minimal.^29^, ^30^ Cervical cancer prevention efforts are reduced further in the COVID‐19 era.^31^, ^32^ Even when vaccination reaches reasonable coverage levels among young women, older unvaccinated birth cohorts will remain at risk making secondary prevention efforts essential over the coming decades.^33^ WHO recommends either “screen‐and‐treat” using primary HPV screening and ablation/excision or “screen‐triage‐treat” using primary HPV screening followed by visual inspection to choose between ablation/excision. However, treating all HPV‐positive women is likely to lead to overtreatment where HPV prevalence is especially high (eg, sub‐Saharan Africa). Using extended HPV genotyping, particularly separating HPV16‐related HPV types (ie, HPV31, HPV33, HPV35, HPV52, HPV58) from the other HR types (ie, HPV39, HPV59, HPV68, HPV51, HPV56), could achieve significant risk‐stratification for risk‐based management. Moreover, relatively faster detection of HPV16, HPV18/45 and HPV31/33/35/52/58 compared to HPV39/51/56/59/68, and higher viral load compared to lower viral load specimens by the assay, would help, if desired and indicated in the label, prioritize the highest risk women first for management or secondary triage.^27^, ^34^

As a result of this satisfactory pilot evaluation, the ScreenFire HPV assay is being evaluated in two independent US laboratories, for clinical accuracy against both prior HPV reference testing and histopathologic outcome, based on an extensively‐validated set of >2000 provider‐collected cervical samples.^35^

## 5 CONCLUSIONS

In conclusion, the redesigned ScreenFire HPV assay was shown to provide accurate risk‐based genotype grouping. If the ongoing large validation study in two independent laboratories yields similar results, the assay using self‐collected dry swabs will be deployed for field evaluation in multiple international cervical cancer screening sites. The longer‐range public health goal is implementation in resource‐limited settings.

## AUTHOR CONTRIBUTIONS

The work reported in the article has been performed by the authors, unless clearly specified in the text. Conceptualization, Mark Schiffman, Silvia de Sanjose, Kanan T. Desai; methodology, Mark Schiffman, Kanan T. Desai, Silvia de Sanjose, Didem Egemen; formal analysis, Kanan T. Desai, Mark Schiffman, Didem Egemen; Itoju field investigation, Mark Schiffman, Kayode O. Ajenifuja, Clement A. Adepiti, Julia C. Gage, Nicolas Wentzensen, Robert D. Burk; original PCR and Luminex testing, Robert D. Burk; resources, Mark Schiffman, Kayode O. Ajenifuja, Clement A. Adepiti, Nicolas Wentzensen; data curation, Kayode O. Ajenifuja, Julia C. Gage, Clement A. Adepiti, Kanan T. Desai; writing—original draft preparation, Kanan T. Desai; writing—review and editing, all. All authors have read and agreed to the published version of the article.

## CONFLICT OF INTEREST

The authors declare no conflict of interest. Atila BioSystems had no role in the design, analysis, interpretation or drafting of this article.

## ETHICS STATEMENT

Both Nigerian and National Cancer Institute (NCI, US) institutional review boards (IRBs) (NCT 00804466) approved the original study protocol that included consent for specimen storage for future research.

## Data Availability

The data that support the findings of our study are available from the corresponding author upon reasonable request.
